# A selective CB2R agonist (JWH133) protects against pulmonary fibrosis through inhibiting FAK/ERK/S100A4 signaling pathways

**DOI:** 10.1186/s12890-023-02747-3

**Published:** 2023-11-13

**Authors:** Xiao Wu, Lina Chen, Yiju Cheng, Yuquan Zhang, Wenting Yang, Lin Pan, Chenkun Fu, Honglan Zhu, Menglin Zhang

**Affiliations:** 1 Department of Critical Care Medicine, The Second People’s Hospital of Guiyang, Guiyang, 550023 People’s Republic of China; 2 Guiyang Public Health Clinical Center, Guiyang, 550004 People’s Republic of China; 3 Department of Respiratory and Critical Care Medicine, The First People’s Hospital of Guiyang, Guiyang, 550004 People’s Republic of China; 4 Guizhou Medical University, Guiyang, 550004 People’s Republic of China; 5 Department of Respiratory and Critical Care Medicine, The Affiliated Hospital of Guizhou Medical University, Guiyang, 550004 People’s Republic of China

**Keywords:** Pulmonary fibrosis, Bleomycin, Cannabinoid receptor type 2, JWH133

## Abstract

**Background:**

The combination of the endocannabinoid system (ECS) and the type 2 cannabinoid receptor (CB2R) can activate various signal pathways, leading to distinct pathophysiological roles. This interaction has gained significant attention in recent research on fibrosis diseases. Focal adhesion kinase (FAK) plays a crucial role in regulating signals from growth factor receptors and Integrins. It is also involved in the transformation of fibroblasts into myofibroblasts. This study aims to investigate the impact of the CB2R agonist JWH133 on lung fibrosis and its potential to alleviate pulmonary fibrosis in mice through the FAK pathway.

**Methods:**

The C57 mice were categorized into five groups: control, BLM, BLM + JWH133, BLM + JWH133 + NC, and BLM + JWH133 + FAK groups.JWH133 was administered to mice individually or in conjunction with the FAK vector. After 21 days, pathological changes in mouse lung tissues, inflammatory factor levels, hydroxyproline levels, and collagen contents were evaluated. Moreover, the levels of the FAK/ERK/S100A4 pathway-related proteins were measured.

**Results:**

JWH133 treatment decreased inflammatory factor levels, attenuated pathological changes, and reduced extracellular matrix accumulation in the mouse model of bleomycin-induced pulmonary fibrosis; however, these effects were reversed by FAK. JWH133 attenuated fibrosis by regulating the FAK/ERK/S100A4 pathway.

**Conclusions:**

The results presented in this study show that JWH133 exerts a protective effect against pulmonary fibrosis by inhibiting the FAK/ERK/S100A4 pathway.Therefore, JWH133 holds promise as a potential therapeutic target for pulmonary fibrosis.

**Supplementary Information:**

The online version contains supplementary material available at 10.1186/s12890-023-02747-3.

## Introduction

Idiopathic pulmonary fibrosis(IPF) refers to a chronic interstitial lung disease of unclear etiology. It manifests as progressive dyspnea, with a median survival of 2–5 years from diagnosis [1]. The disease is characterized by persistent tissue injury that triggers excessive activation of myofibroblasts; rapid proliferation, migration, and secretion of collagen type I (Col-I); as well as the formation of contractile organelles, including α-smooth muscle actin (α-SMA), causing abnormal accumulation of extracellular matrix (ECM) [2]. Nintedanib and pirfenidone are currently used in treating idiopathic pulmonary fibrosis; however, their efficacy is limited, with many patients progressing to end-stage disease [3]. Therefore, developing effective antifibrotic drugs is warranted to impede the fibrotic response and improve survival in such patients.

The endocannabinoid system (ECS) combines endocannabinoids, cannabinoid receptors, and related enzymes. The system includes the cannabinoid type 1 receptor (CB1R) and cannabinoid type 2 receptor (CB2R), which are engaged in signal transfer through cannabinoids [4]. CB1R is generally denoted in the nervous system, whereas CB2R is mostly expressed in peripheral organs with immune functions, such as the thymus, lung, spleen, and liver [5, 6]. Several studies have concentrated on the role of cannabinoid receptors in the nervous system, with recent ones suggesting that the ECS with immunomodulatory functions serves as a target in treating inflammatory and fibrotic diseases. For example, in bleomycin (BLM)-induced mouse models of skin fibrosis, CB2R agonist inhibited collagen synthesis, delayed ECM deposition, and prevented fibrotic progression in vitro and in vivo, without psychoactive side effects [7]. Treatment with the CB2R agonist AM1241 reduced oxidative stress and inflammation and improved myocardial fibrosis [8]. Moreover, CB2R agonist reduced nicotine-induced interstitial pulmonary fibrosis, indicating its antifibrotic action [9].

JWH133 is a potent CB2R-selective agonist that binds to CB2R with a 200-fold greater affinity than CB1R. We used JWH133 and focal adhesion kinase (FAK) in treating pulmonary fibrosis in BLM-induced mouse models of pulmonary fibrosis. To examine the impact of JWH133, we assessed alterations in lung tissue and signaling pathway related proteins following JWH133 treatment. Moreover, we examined the underlying mechanisms.

## Methods

### Animal study

Animal experiments were approved by the Ethics Committee of Guizhou Medical University (Certificate No. SYXK [Qian] 2018–0001). In accordance with the Regulations for the Administration of Affairs Concerning Experimental Animals in China, the experiments were conducted. Totally 30 specific-pathogen-free (SPF) C57BL/6J healthy male mice (8 weeks, weighing 20–23 g) were procured from the Animal Experiment Center of Guizhou Medical University. Then, the mice were housed at 20–25 °C, a relative humidity of 45–60%, and a light/dark cycle of 12:12 h, with free access to water and food. After a week of acclimation, the mice were randomized into five groups: control, BLM, BLM + JWH133, BLM + JWH133 + NC, and BLM + JWH133 + FAK groups. To establish a fibrotic mouse model, the mice were anesthetized (1% pentobarbital sodium, 40 mg/kg) and positioned supine.Subsequently, BLM was administered into the trachea at a dose of 0.5 mg/100 g (MCE, New Jersey, USA). In contrast, the control group was subject to injection with the same volume of saline. On day one after BLM induction, the mice in the BLM + JWH133, BLM + JWH133 + NC, and BLM + JWH133 + FAK groups were intraperitoneally injected with 2.5 mg/kg of JWH133(APExBIO, Houston, USA) daily [10]. The mice in the BLM + JWH133 + NC and BLM + JWH133 + FAK groups were additionally administered with intraperitoneal injections of equal amounts of empty viral vectors and FAK adenoviral vectors (Genechem,Shanghai, China).On day 21, Blood samples were collected from the retro-orbital plexus of each mouse, the samples were subjected to biochemical analysis. Afterward, the mice were euthanized through cervical dislocation. The mice were positioned on an anatomical plate, and the tissue was carefully separated layer by layer. The lungs of the mice were removed, and the serum and right lung were frozen at − 80℃ for later use. Additionally, the left lung was fixed with 4% Paraformaldehyde to facilitate pathological diagnosis and immunohistochemical detection.

### Pathological identification

The lung tissues were fixed with 4% paraformaldehyde for 2 days, dehydrated with ethanol, embedded in paraffin, sectioned at the thickness of 3 μm, and exposed to hematoxylin and eosin (HE) and Masson’s staining. Afterward, three pathologists utilized a microscope to select 30 visual fields within each sample for scoring randomly. The average score of all the visual fields was considered as the fibrosis degree score of the sample. The extent of alveolitis (HE staining) and pulmonary fibrosis (Masson’s staining) were assessed using the methods described by Szapiel, S.V. and Ashcroft, T [11, 12].

### Immunofluorescence staining

The paraffined-embedded lung tissue sections were dewaxed and hydrated, treated with sodium citrate for antigen retrieval, immersed in hydrogen peroxide to block the endogenous peroxidase, sealed with 3% bovine serum albumin for half an hour, and exposed to incubation through the night with Col-I (ab270993, Abcam, USA) at 4 °C. Next, the sections were subject to incubation with a secondary antibody (ab6721, Abcam, USA) for 50 min, conventionally dehydrated, cleared, stained using diaminobenzidine, and sealed using neutral resin. Finally, the samples were observed under a microscope, and the collected images were analyzed using Image J to evaluate the mean optical density values of Col-I.

### Enzyme-linked immunosorbent assays

The collected blood samples were subject to centrifugation (2500 × g) at 4℃ for 10 min. Next, we proceeded with the collection of supernatant. Moderately diluted samples and standards were added to the enzyme plate pre-coated with anti-mouse TNF-α monoclonal antibody (4 A Biotechnch Beijing, China). Subsequently, a Biotinylation anti-mouse TNF-α antibody was added, followed by a 2-hour incubation. After washing, Horseradish peroxidase-labeled avidin was added, and then the chromogenic agent was introduced after another round of washing. The mixture was incubated for 15 min, and the reaction was halted with the termination solution. The OD value was measured at 450 nm, and the TNF-α concentration (pg/mL) in the mouse samples was calculated by plotting a standard curve. The experimental steps for IL-6 and IL-1β were identical to those used for TNF-α.

### Measurement of hydroxyproline (HYP) assay

HYP is the main component of collagen, and the collagen content was measured with an HYP kit (Jiancheng Bioengineering Institute, Nanjing, China). Lung tissue was weighed in line with the manufacturer’s guidance, with the HYP levels expressed in µg/mg.

### Western blot

To extract total protein, 20 mg of lung tissue was supplemented to 200 µL of RIPA lysate and centrifuged (12,000 × g, 10 min, 4 °C), followed by protein quantification. Subsequently, the protein samples were separated on sodium dodecyl sulfate-polyacrylamide gel (SDS-PAGE) (Beyotime, Shanghai, China), followed by transfer on polyvinylidene difluoride (Millipore, Billerica, MA, USA) membranes. Additionally, the membranes were blocked with the use of 5% skim milk for 60 min and incubated overnight at 4 °C with primary antibodies, including Col-I (1:1000, a b138492, Abcam, USA), Col-III (1:1000, ab184993, Abcam), α-SMA(1:1000; #19,245, Cell Signaling Technology, USA), ERK1/2 (1:1000; #4695, Cell Signaling Technology, USA), p-ERK1/2(1:2000;#4370, Cell Signaling Technology, USA), FAK(1:1000;#3285, Cell Signaling Technology, USA), p-FAK (1:1000; #3283, Cell Signaling Technology, USA), S100A4 (1:1000; #13,018, Cell Signaling Technology, USA), Subsequently, an HRP-labeled secondary antibody(goat anti-rabbit IgG, #7074, 1:2000; Cell Signaling Technology, USA) was supplemented to the samples, followed by incubation at room temperature for 60 min. Subsequently, the samples were visualized with the use of the electrochemiluminescence (Meilunbio, Dalian, China) method, and the grayscale value was analyzed using Image J. The grayscale value ratio of the target protein to GAPDH was evaluated for the relative expression of the target protein.

### Quantitative real-time polymerase chain reaction

By adopting TRIzol (Absin, Beijing, China), the extraction of total RNA was performed from the lung tissue. Before reverse transcription to cDNA, RNA concentration and purity were determined. Reverse transcription into cDNA based on the guidance of the kit (PrimeScript™ RT reagent Kit, Takara, Shiga, Japan). The CFX Connect™ Real-Time System (Bio-Rad, California, USA) was carried out for 40 cycles of 95°C for 30 s, 95°C for 5 s, and 60°C for 30 s. Primers included: Col-I-forward 5’-GCCAAGAAGACATCCCTGA-3’, reverse 5’-GCATACCTCGGGTTTCCA-3’, Col-III-forward 5’-AAAGAATGGGGAGACTGGA-3’, reverse.

5’-TGCCTTGTAATCCTTGTGGA-3’, α-SMA-forward 5’-GCACCGCAAATGCTTCTA-3’, reverse 5’-TGGTCCAGGGTTTCTTACTCC-3’, GAPDH-forward 5’-GGTTGTCTCCTGCGACTTCA-3’, reverse 5’-TGGTCCAGGGTTTCTTACTCC-3’. With GAPDH as the internal control, the 2-ΔΔCt method was employed to calculate mRNA expression.

### Statistical analysis

Data were explored with the application of GraphPad Prism 9.0 software (GraphPad Software, Inc., La Jolla, CA, USA). In addition, measurement data are denoted to be mean ± standard deviation. Based on the student’s *t*-test or one-way analysis of variance, comparisons between groups were performed. In addition, all the experiments were conducted in triplicates. *P* < 0.05 was thought to be of statistical significance.

## Results

### Pathological changes of lung tissue in mice administered with JWH133

**Fig. 1:**
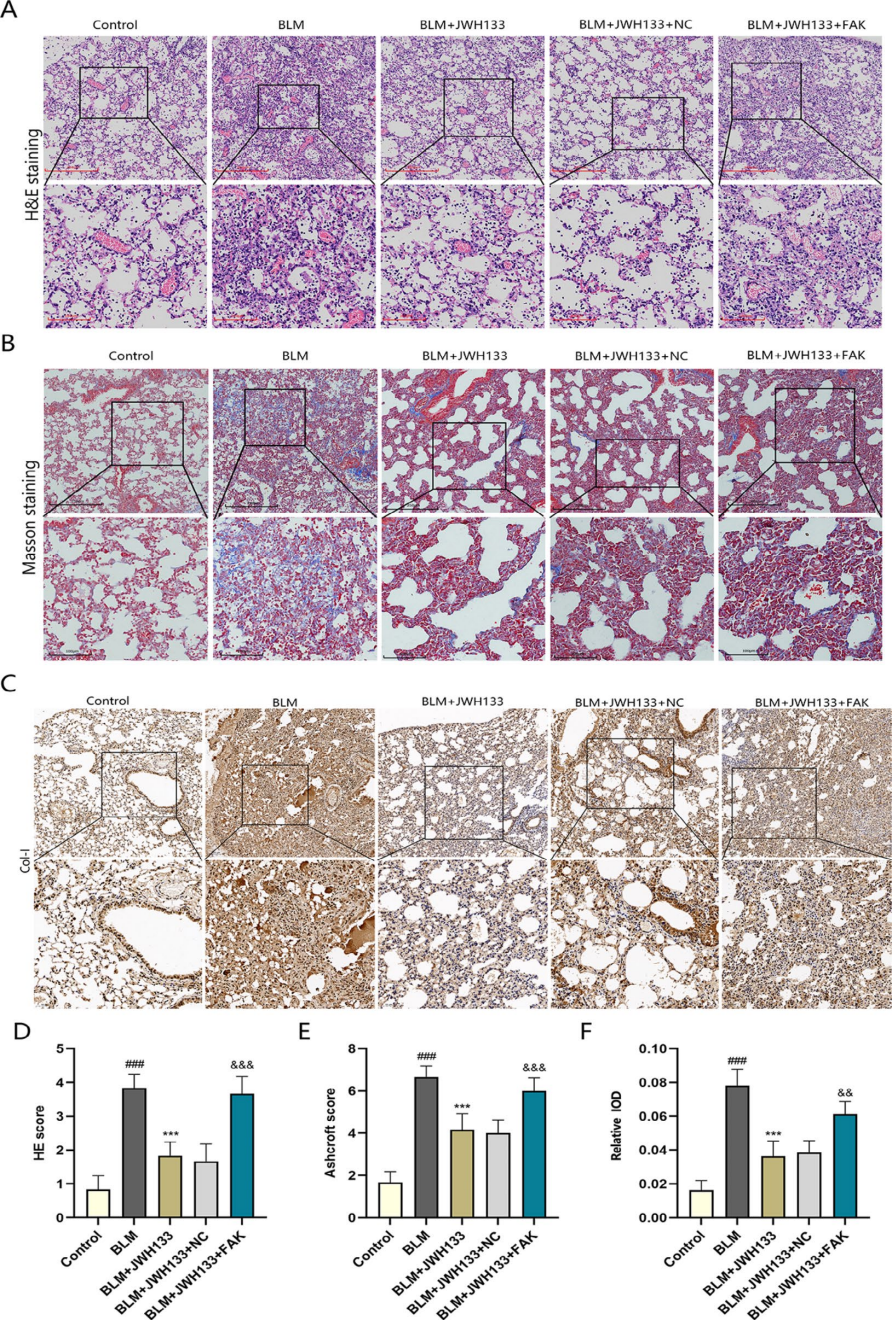
Pathological changes of lung tissue in mice treated with JWH133. **(A)** H&E staining of mouse-lung tissue in control.Scale bar: 250 μm and 100 μm. **(B)** Masson staining of mouse-lung tissue in control.Scale bar: 250 μm and 100 μm. **(C)** Immunohistochemistry images of mouse-lung tissue in control.Scale bar: 100 μm and 50 μm **(D)** HE staining score for each group.n = 6. **(E)** Ashcroft score for each group.n = 6. **(F)** Relative IOD for each group.n = 5.^###^*P* < 0.001 vs. control group;^***^P < 0.001 vs. model group; ^&&^*P* < 0.01,^&&&^*P* < 0.001 vs. BLM + JWH133 + NC.

In pulmonary fibrosis, ECM overproduction and accumulation occur in a disorderly manner, thereby impairing organ function and causing lung tissue remodeling. In this study, JWH133 reduced collagen synthesis in mice with pulmonary fibrosis through CB2R. As shown in Fig. 1A illustrates that the HE staining displayed intact lung tissue structure in the control mice, with no signs of interstitial exudation, swelling, or inflammatory cell infiltration. Significant destruction of alveolar structure, thickened alveolar septum, inflammatory cell infiltration, and obvious fibroplasia were found in the BLM group. The BLM + JWH133 and BLM + JWH133 + NC groups exhibited improved alveolar structure and reduced inflammatory cell infiltration, whereas the BLM + JWH133 + FAK group exhibited aggravated lung tissue destruction because of FAK treatment. In control mice, Masson’s staining revealed slight blue collagen deposition around blood vessels and trachea. The BLM group exhibited a loss of alveolar structure in the lung tissue, solid lung changes, large blue staining areas in the interstitium, and increased collagen deposition. Blue collagen decreased in the lung tissue after the JWH133 intervention; however, treatment with FAK increased blue collagen levels in the BLM + JWH133 + FAK group (Fig. 1B). In addition, alveolitis (Fig. 1D) and fibrosis (Fig. 1E) were significantly reduced in the BLM + JWH133 group; however, an increase in alveolitis and fibrosis was observed in the BLM + JWH133 + FAK group. In the immunohistochemical experiments (Fig. 1C), the formation of the Col-I-positive product was observed, which appeared as a brownish-yellow color. The mean optical density values (Fig. 1F) and Col-I positive expression increased in the BLM group; however, a decrease in these values was observed in the BLM + JWH133 and BLM + JWH133 + NC groups. Increased Col-I deposition and mean optical density values were observed after FAK overexpression.

### JWH133 alleviates pulmonary inflammatory response in mice

**Fig. 2:**
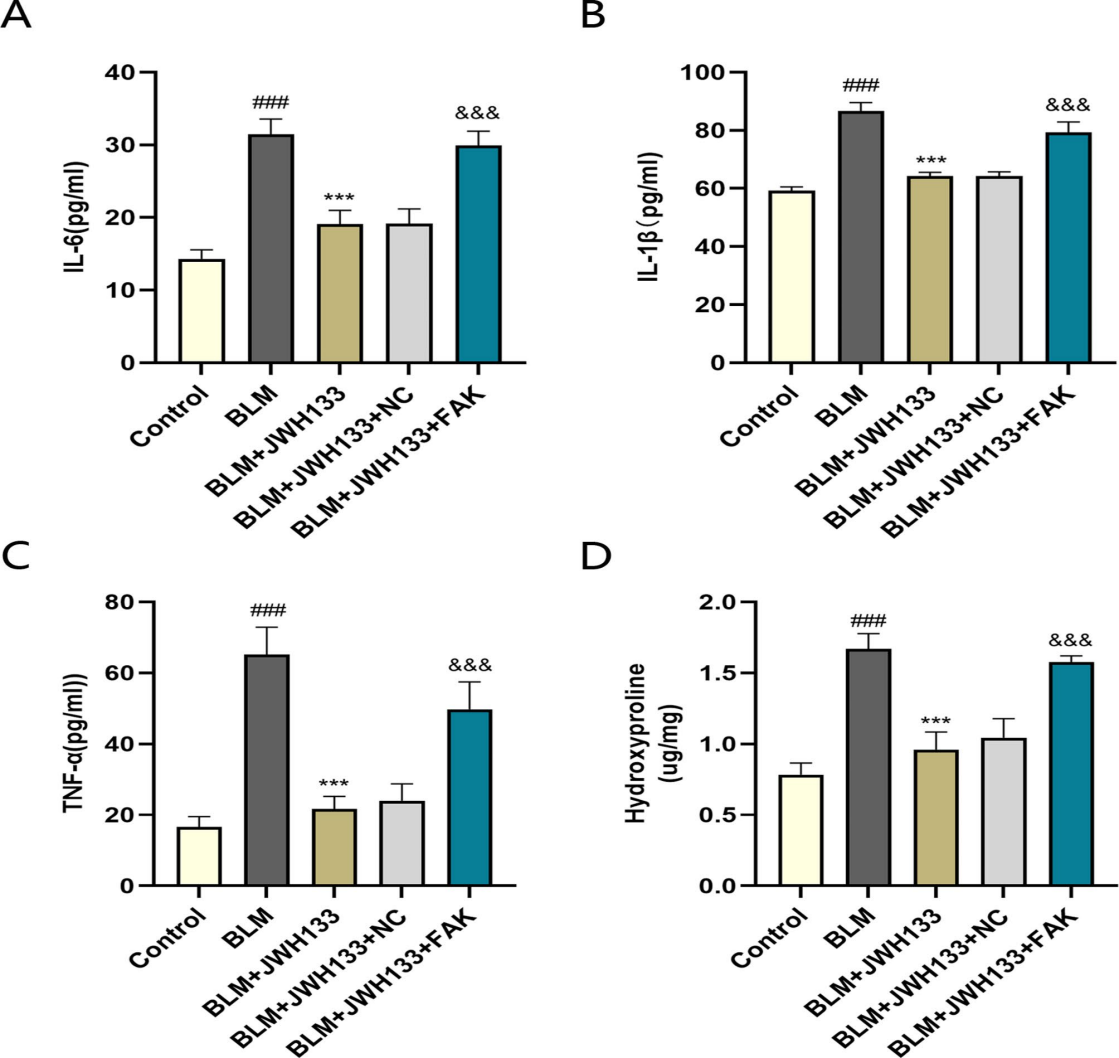
JWH133 alleviates pulmonary inflammatory response in mice with BLM .The levels of inflammatory factors IL-6 **(A)** 、IL-1β **(B)** and TNF-α **(C)** in serum of lung tissue were measured by enzyme-linked immunosorbent assay (ELISA).n = 6. **(D)** The content of hydroxyproline in lung tissue.n = 6.^###^*P* < 0.001 vs. control group;^***^P < 0.001 vs. model group;^&&&^*P* < 0.001 vs. BLM + JWH133 + NC.

JWH133 activated CB2R regulating the inflammatory response, and subsequently reduced pulmonary fibrosis in mice.The ELISA results showed significantly higher levels of inflammatory markers TNF-α, IL-6, and IL-1β in the serum of the BLM group (Fig. 2A-C), which decreased after the administration of JWH133. By contrast, inflammatory factor levels increased after FAK overexpression. High HYP content was observed in the lung tissue of the BLM group than in that of the control group, indicating an increase in collagen content. However, HYP content decreased after JWH133 intervention, with the effect being reversed after treatment with FAK(Fig. 2D).

### JWH133 decreased the expression of fibrosis proteins induced by bleomycin (BLM) in mice

**Fig. 3:**
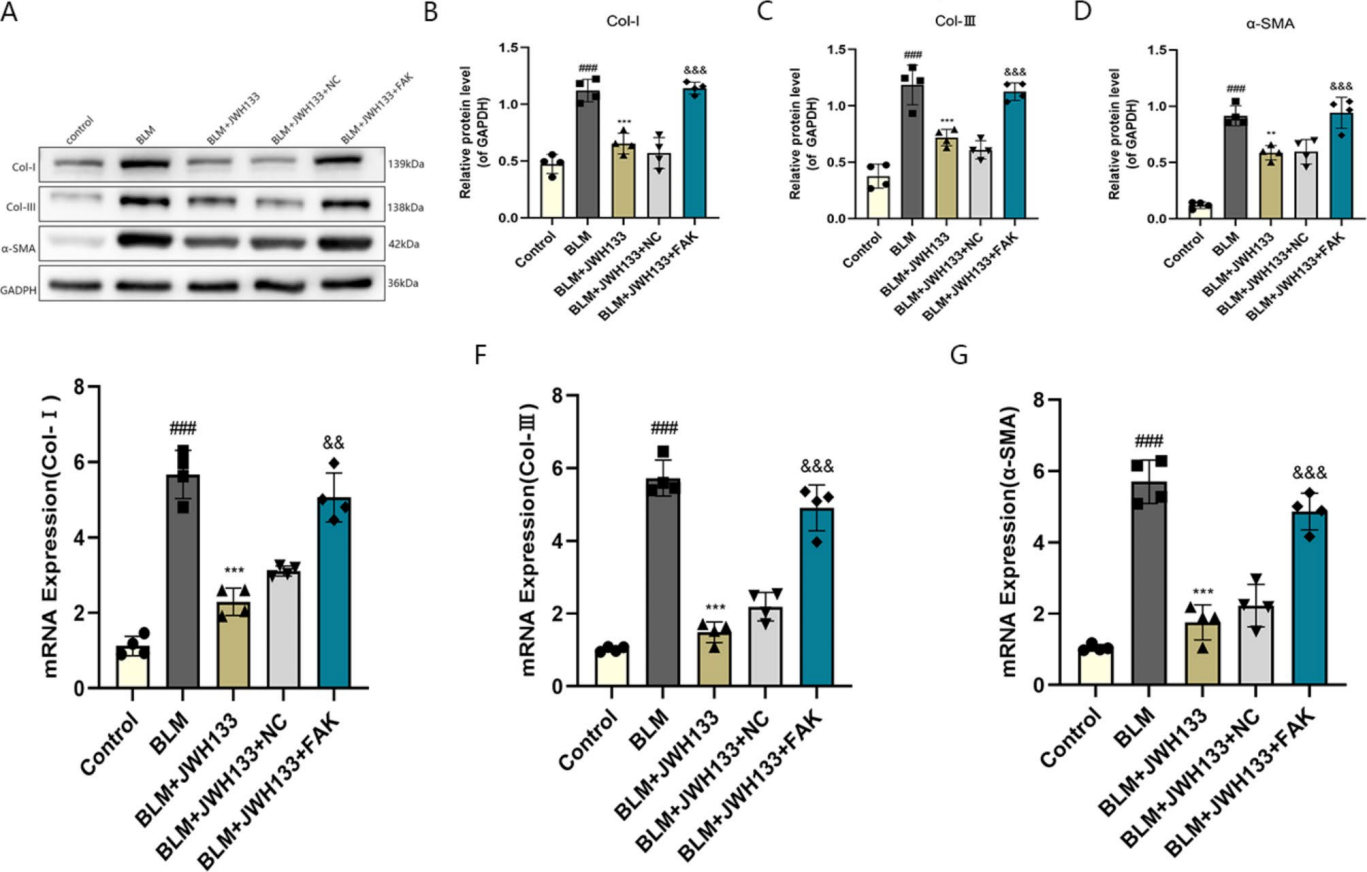
JWH133 decreased the expression of fibrosis proteins induced by bleomycin (BLM) in mice. **(A)** Protein expression of Col-I, Col-III and α-SMA, in lung tissues of mice was measured by Western blot. Relative protein expression of **(B)** Col-I, **(C)** Col-III,and **(D)** α-SMA were quantified by normalizing to GAPDH.Real-time PCR analyses of **(E)** Col-I, **(F)** Col-III and **(G)** α-SMA in lung tissues. GAPDH was used as the invariant control for calculating fold changes in mRNA levels.Data were expressed as mean ± SEM, and statistical analysis was conducted using the one-way ANOVA with the Tukey test.^###^*P* < 0.001 vs. control group;^**^P < 0.01,^***^P < 0.001 vs. model group;^&&^*P* < 0.01,^&&&^*P* < 0.001 vs. BLM + JWH133 + NC.n = 4

As shown in Fig. 3A–D, Western blotting demonstrated increased protein levels of Col-I, Col-III, and α-SMA in the BLM group. However, after the intervention of JWH133, these levels were reduced in the BLM + JWH133 and BLM + JWH133 + NC groups.Nonetheless, the administration of FAK reversed this effect. RT-qPCR analysis also revealed that JWH133 effectively inhibited the up-regulation of these fibrosis markers, including Col-I, Col-III, and α-SMA in mice with BLM-induced pulmonary fibrosis in mice(Fig. 3E–G). Additionally, similar to BLM, the overexpression of FAK induced an up-regulation of these fibrosis markers. These findings suggest that JWH133 has a protective impact on BLM-induced pulmonary fibrosis in mice.

### JWH133 prevented pulmonary fibrosis by suppressing FAK/ERK/S100A4 signaling pathway

**Fig. 4:**
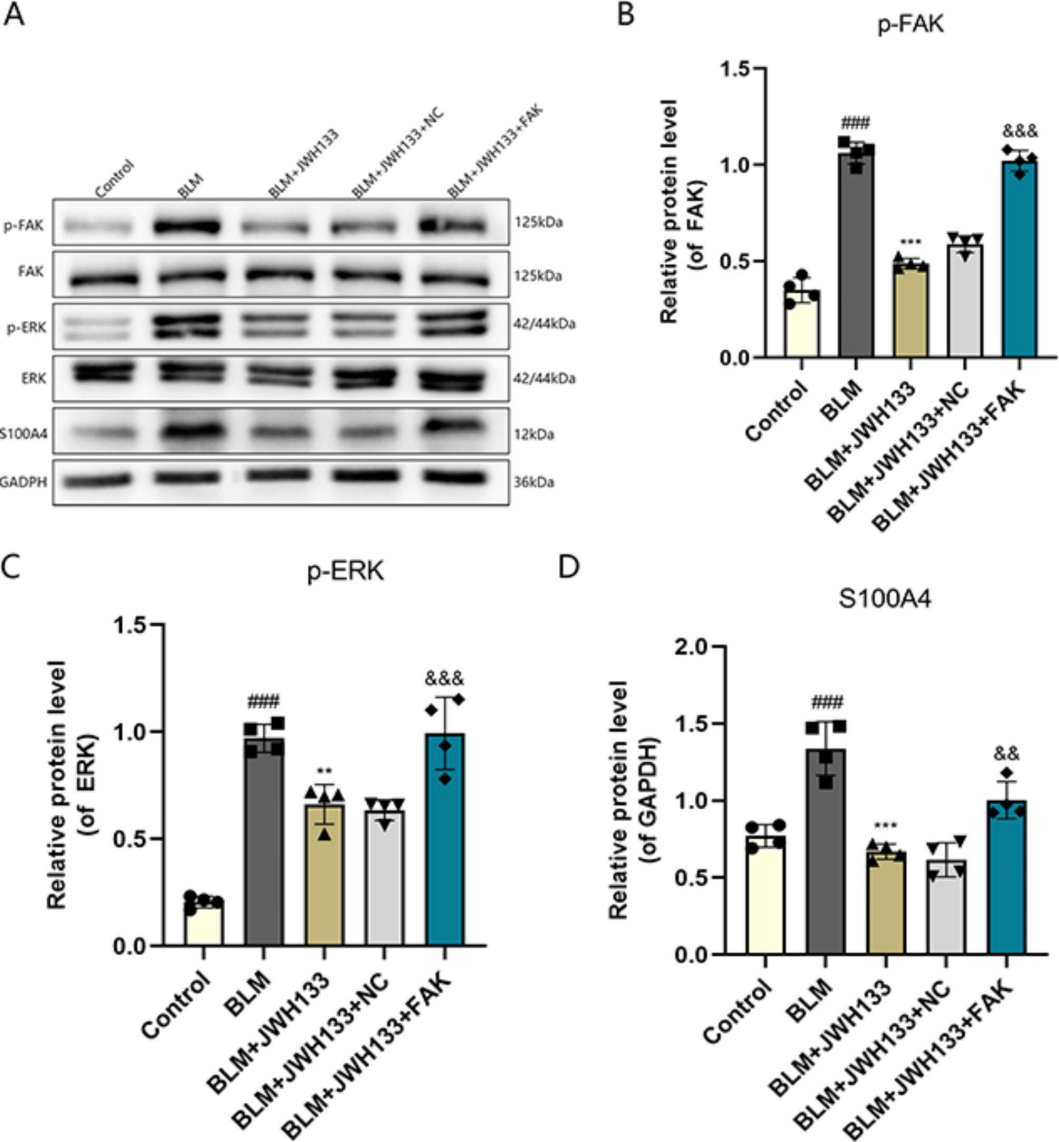
JWH133 prevented pulmonary fibrosis by suppressing FAK/ERK/S100A4 signaling pathway. **(A)** Protein expression of p-FAK,FAK,p-ERK,ERK,S100A4 in lung tissue were detected by Western blot. Relative protein expression of **(B)** p-FAK, **(C)** p-ERK, and **(D)** S100A4 were quantified by normalizing to GAPDH.Data were expressed as mean ± SEM, and multiple comparisons were performed using one-way ANOVA with the Tukey test.^###^*P* < 0.001 vs. control group;^**^P < 0.01,^***^P < 0.001 vs. model group;^&&^*P* < 0.01,^&&&^*P* < 0.001 vs. BLM + JWH133 + NC.n = 4

Western blotting was adopted to further understand the antifibrotic mechanism of JWH133 in mouse lungs. The results indicated an obvious increase in the protein levels of p-FAK, p-ERK, and S100A4 in lung tissues of the BLM group. The levels decreased after the JWH133 intervention. To overexpress FAK in BLM-induced mice, we administered FAK adenovirus. Consequently, we observed an up-regulation of p-FAK, p-ERK, and S100A4(Fig. 4A-D).Moreover, the overexpression of FAK hindered the inhibitory effects of JWH133 on BLM-induced pulmonary fibrosis in mice. In summary, JWH133 ameliorates the symptoms of pulmonary fibrosis in mice through the FAK/ERK/S100A4 pathway.

## Discussion

Our results indicated that CB2R activation exerted antifibrotic effects in vivo, and JWH133 effectively prevented BLM-induced pulmonary fibrosis in mice through the FAK/ERK/S100A4 pathway.

BLM is a drug widely used to induce pulmonary fibrosis in animal models [13]. In this study, histopathological examination using Masson’s staining revealed that BLM-induced mice exhibited severe collagen deposition. Increased Col-I deposition, as revealed in immunohistochemical experiments, and elevated collagen levels were indirectly reflected in the HYP measurements, thereby validating our animal model. After the JWH133 intervention, the mice exhibited improvement in the aforementioned pathological examinations; moreover, reduced mRNA and protein levels of the relevant indicators of pulmonary fibrosis activation, such as Col-I, Col-III, and α-SMA, were observed. In addition, the obtained findings indicated that JWH133 enhanced ECM deposition. JWH133 reduced α-SMA expression and collagen content, thereby producing an antifibrotic effect [14]. However, FAK overexpression enhanced the aforementioned indicators; the obtained findings are consistence with that of Correia-Sá I et al. Injury is crucial for the initiation of fibrotic processes, and sustained inflammation is essential to activate wound healing and to initiate fibrosis; therefore, the fibrotic process is regulated to some extent by inflammatory mediators [15]. Wang et al [16]. studied skin wound treatment and evidenced that CB2 agonists exerted an antifibrotic effect by downregulating the levels of pro-inflammatory cytokines IL-1β, IL-6, and TNF-α. Additionally, we also found increased levels of inflammatory indexes TNF-α, IL-6, and IL-1β in the BLM group; however, the levels reduced after JWH133 treatment. HE staining confirmed that cannabinoids inhibited the fibrotic process by controlling the inflammatory response and by reducing collagen synthesis.

Focal adhesion kinase (FAK) refers to a non-receptor protein tyrosine kinase with a molecular weight of 125 kDa [17]. It plays a key role in the integrin receptor on the cell surface to mediate the cell and ECM proteins around the cell, such as cell migration and proliferation [18, 19]. FAK has six phosphorylation sites, of which tyrosine 397 (Tyr397) is the main autophosphorylation site [20]. Through the activation of this site, FAK plays a key role in tissue remodeling and regulates cell shape, function, survival, and cell-matrix signaling [21]. Moreover, FAK binds to the SH2 structural domain of the Src family and activates other phosphorylation sites to trigger the activation of the FAK downstream Ras/Raf/MEK/ERK pathway [22, 23]. As a member of the mitogen-activated protein kinase (MAPK) family (ERK1/ERK2, also known as p44/42 MAPK), an extracellular signal-regulated kinase (ERK) is a signal transduction protein that transmits mitogenic signals [24]. Studies have demonstrated that the CB2R agonist JWH133 exerted antifibrotic effects in mice. JWH133 inhibited the TGF-β1-induced ERK1/2 pathway in human conjunctival capsule fibroblasts to improve ECM deposition. Therefore, we hypothesized that JWH133 could inhibit the expression and secretion of p-FAK protein and its downstream signal pathway p-ERK protein after activating CB2R. Our results showed that in BLM-induced pulmonary fibrosis in mice, FAK was activated by binding to the integrin of extracellular matrix protein, resulting in an increase in the phosphorylation of Tyr397 of FAK, followed by activation of pathways, including ERK1/2, and an elevation in the level of p-FAK and p-ERK protein was found. However, after treatment with JWH133, the activation of FAK was prevented, and the expression of ERK1/2 was decreased. After overexpression of FAK, it was observed that JWH133 blocked the inhibition of BLM-induced pulmonary fibrosis in mice. These data indicate that JWH133 can inhibit integrin-mediated FAK activation, block FAK-mediated cell migration and proliferation, and protect bleomycin-induced pulmonary fibrosis in mice.

S100A4 is an important member of the S100 calcium-binding protein family and a cell migration regulatory protein. S100A4’s function is mainly to promote inflammation and metastasis [25]. Zhang et al [26]. confirmed that S100A4, secreted by M2-polarized alveolar macrophages, induces α-SMA and Col-I levels, promotes the transformation of lung fibroblasts into myofibroblasts, and synthesizes high levels of ECM components. In this study, an obvious elevation in S100A4 level was found in mice with BLM-induced pulmonary fibrosis. The level reduced after JWH133 treatment and increased after FAK overexpression. These data indicate that JWH133 can reduce the activation of FAK and inhibit the expression and secretion of S100A4 protein, a signal molecule of cell migration cascade, after activating CB2R. Therefore, on the basis of the above-mentioned analysis, it can be preliminarily shown that the FAK/ERK/S100A4 signal pathway may become the vital medium for JWH133 to exert the anti-fibrosis role in mouse pulmonary fibrosis.

In the future, further research is needed to prove whether JWH133 regulates other fibrogenic pathways, such as epithelial-mesenchymal transition(EMT) and transforming growth factor-β(TGF-β) activation to play its role, the safety of JWH133, and the preventive effect of JWH133 in the early phase of fibrosis, but the role of JWH133 in the late stage of fibrosis needs further research.

In this study, CB2R activation by the selective agonist JWH133 ameliorated BLM-induced pulmonary fibrosis in mice through decreasing pulmonary fibrosis score and inhibiting inflammation-associated cytokines IL-6, TNF-α, and IL-1β as well as through decreasing Col-I, Col-III, α-SMA, and HYP levels possibly via hindering the FAK/ERK/S100A4 pathway.

### Electronic supplementary material

Below is the link to the electronic supplementary material.

Supplementary Material 1

## Data Availability

The datasets used and analyzed during the current study are available from the corresponding author on reasonable request.
