# Supplementary material for: A selective CB2R agonist (JWH133) protects against pulmonary fibrosis through inhibiting FAK/ERK/S100A4 signaling pathways
Source: BMC Pulm Med. 2023 Nov 13;23:440. doi: 10.1186/s12890-023-02747-3 (PMC10641936; doi:10.1186/s12890-023-02747-3)
Supplement: Supplementary file 1 — Supplementary Material 1 [file 12890_2023_2747_MOESM1_ESM.pdf]

Figure 3 Col-I

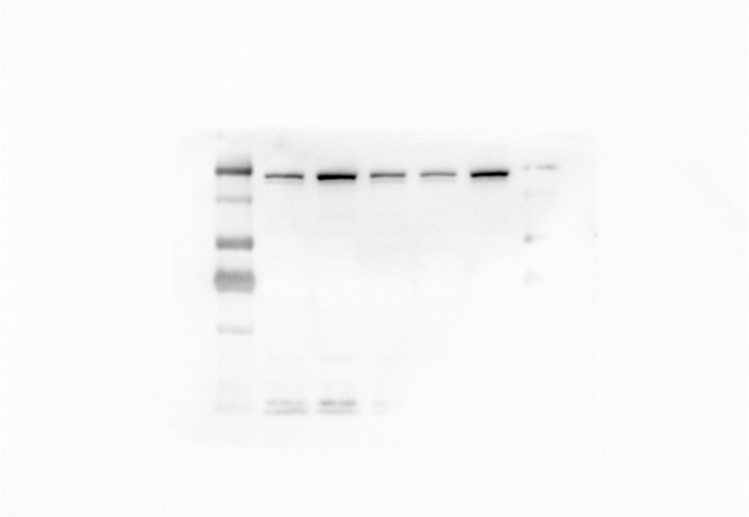

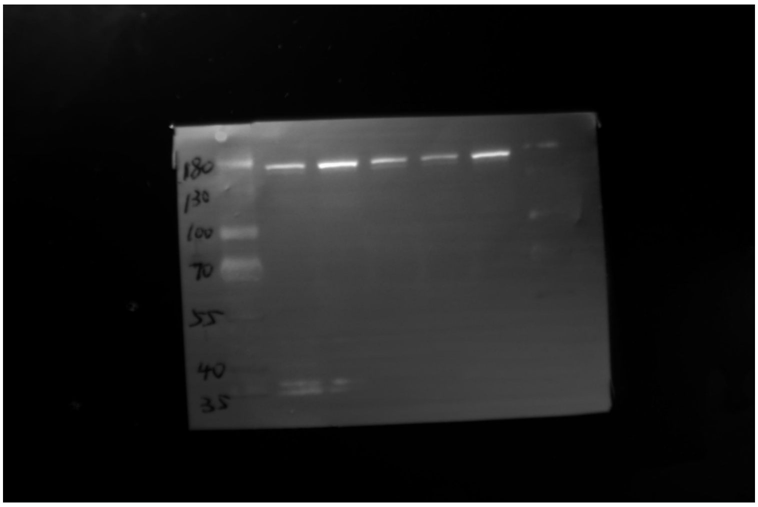

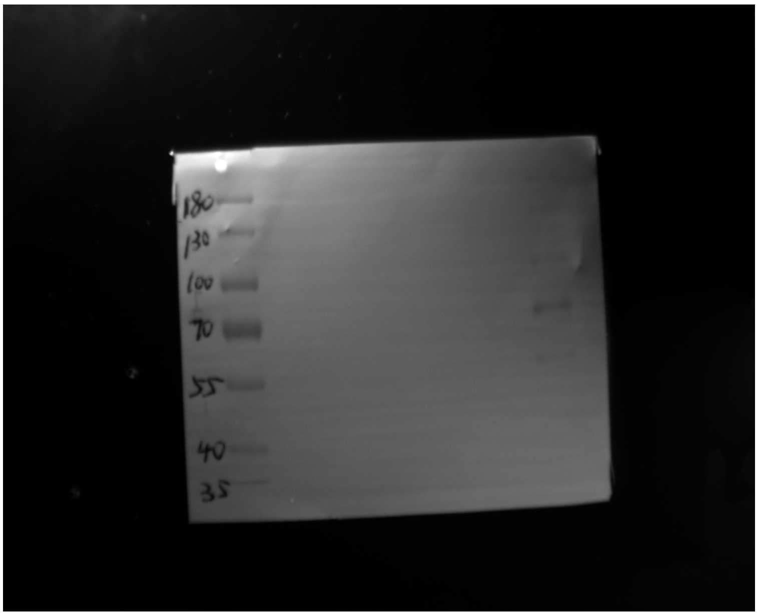

Figure 3 Col-III

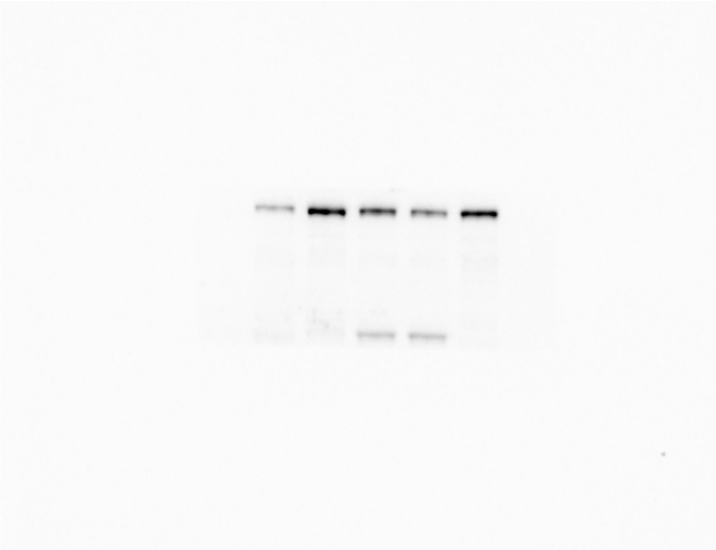

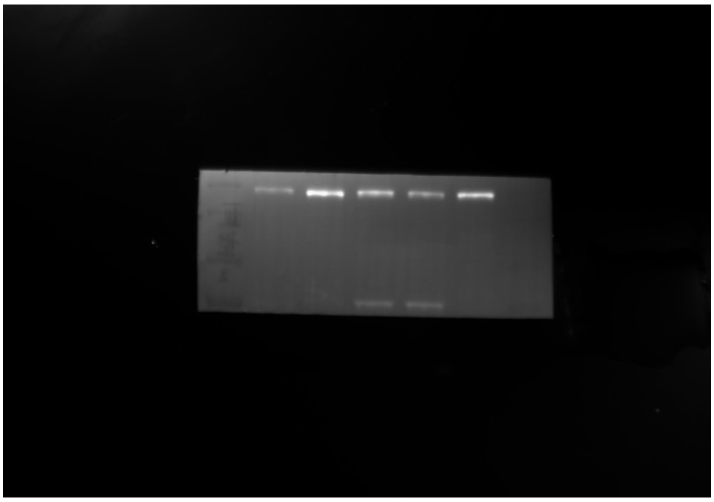

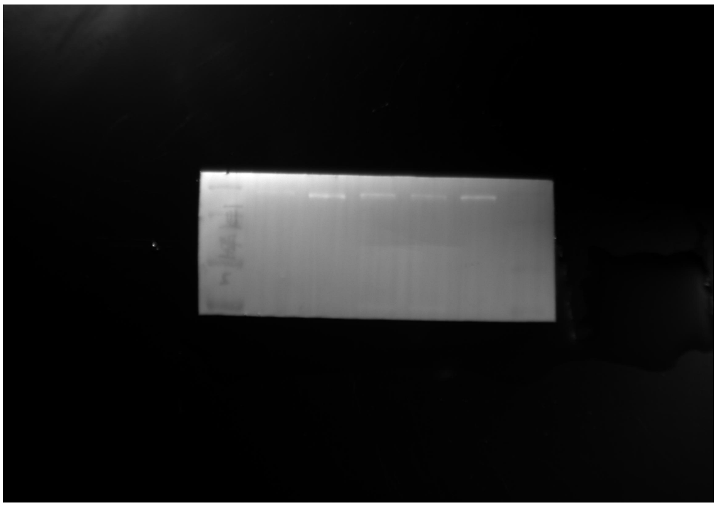

Figure 3 GAPDH

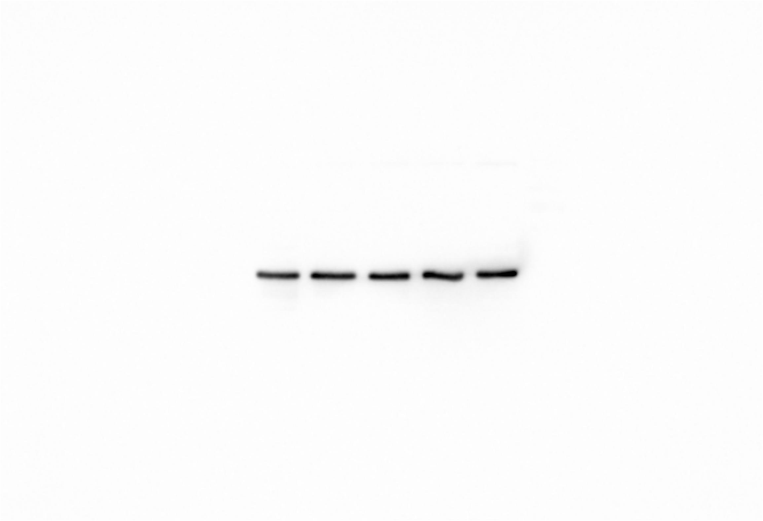

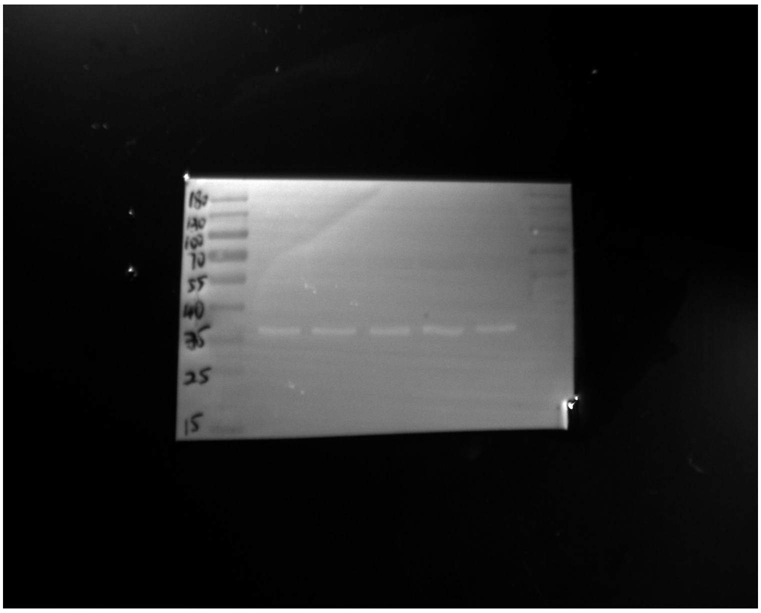

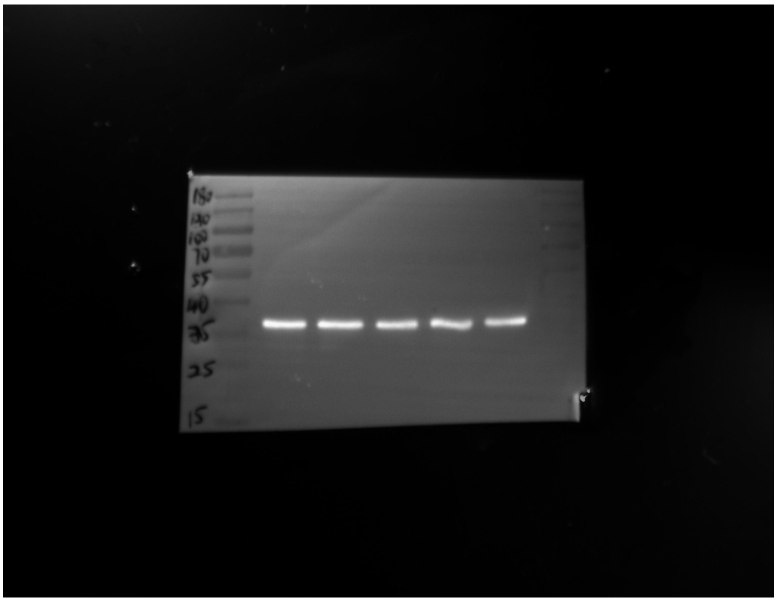

Figure 3 α-SMA

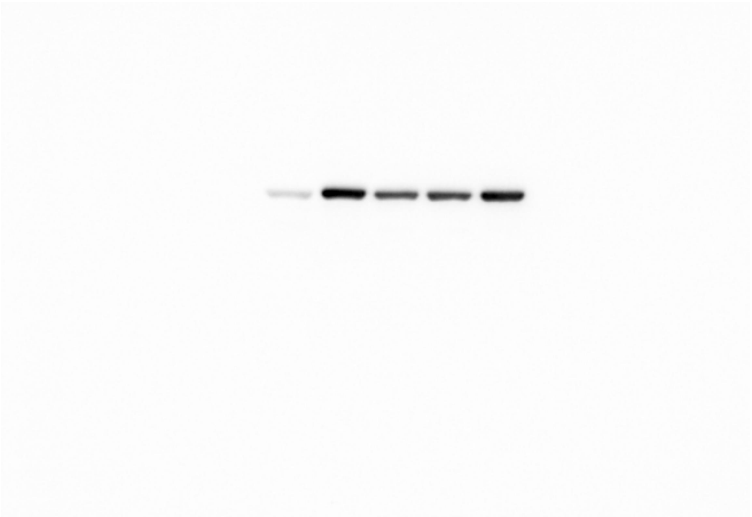

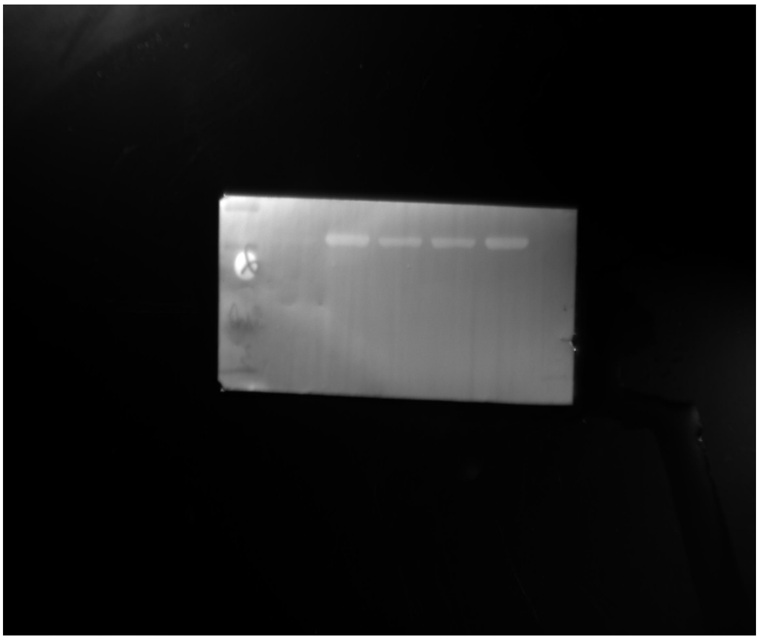

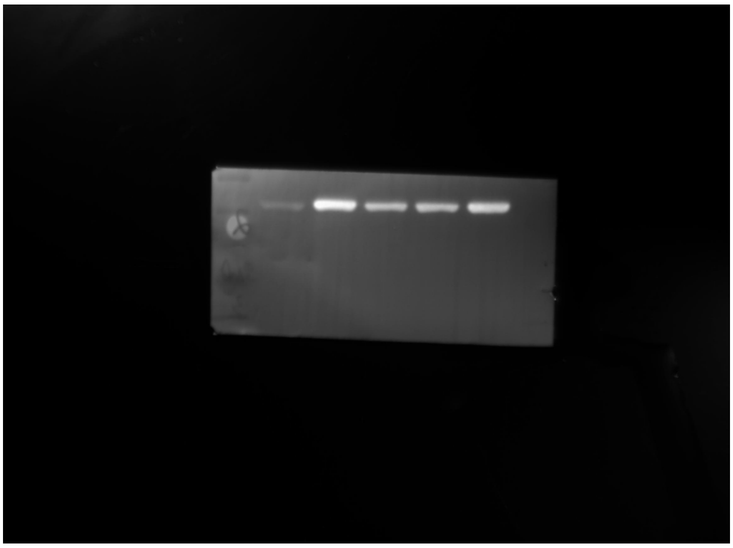

Figure 4 p-FAK

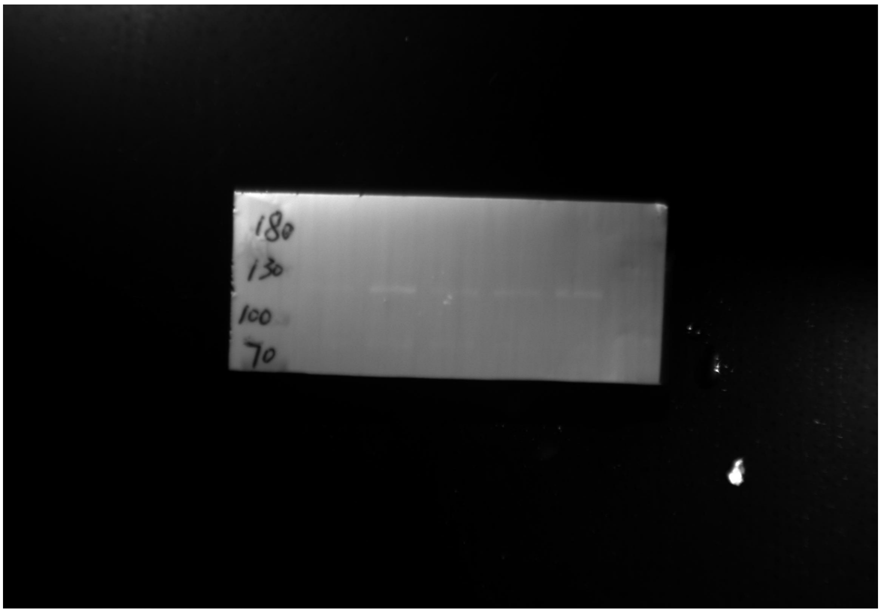

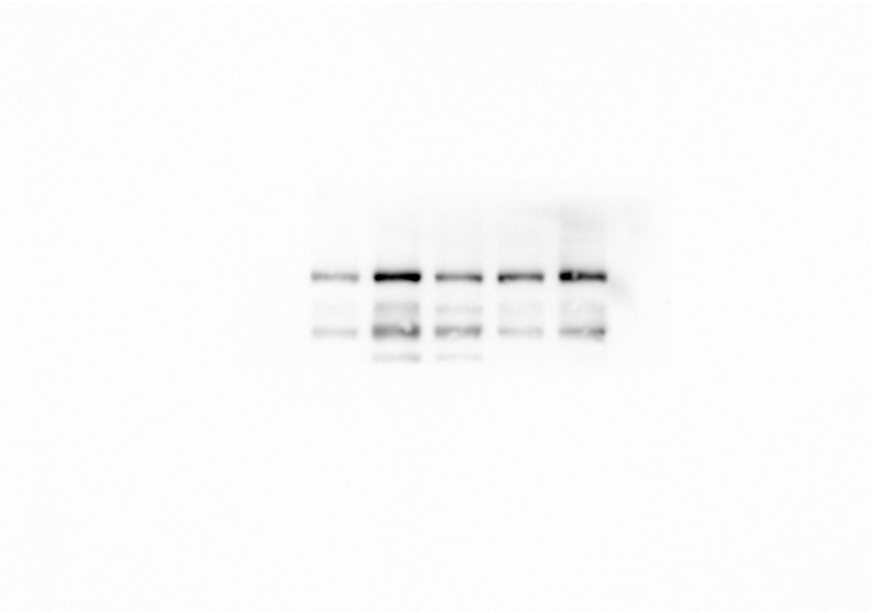

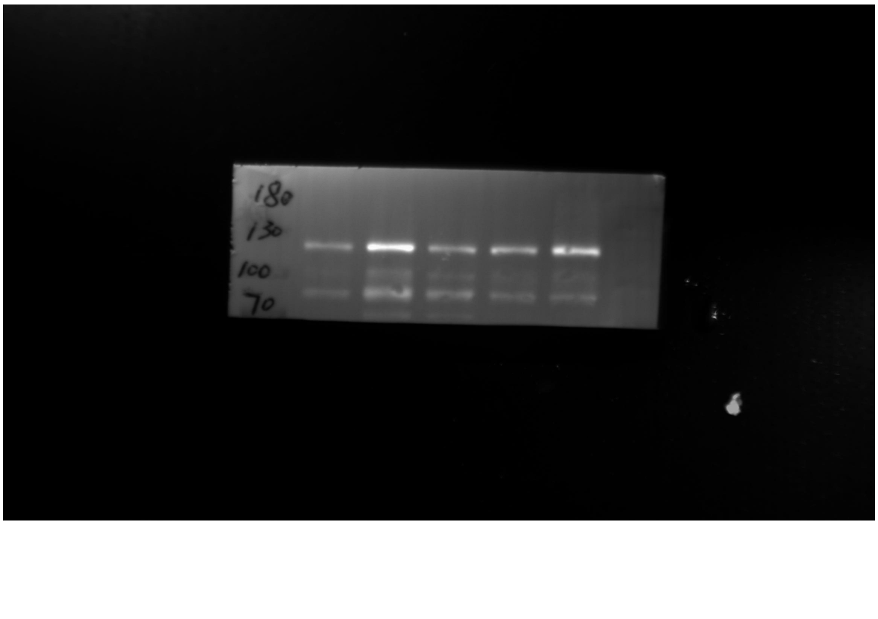

Figure 4　FAK

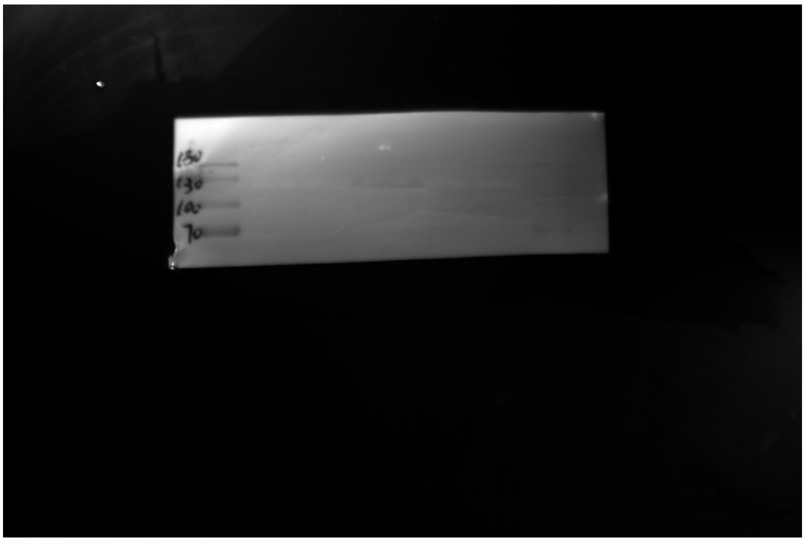

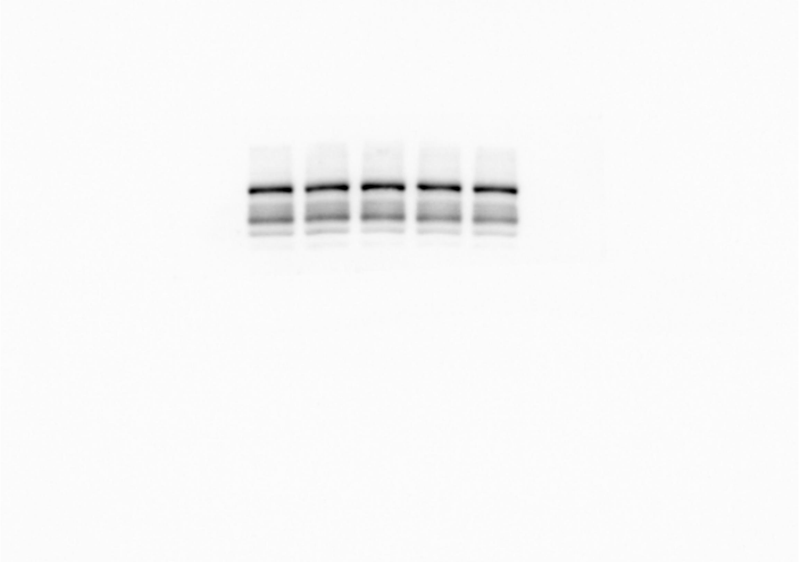

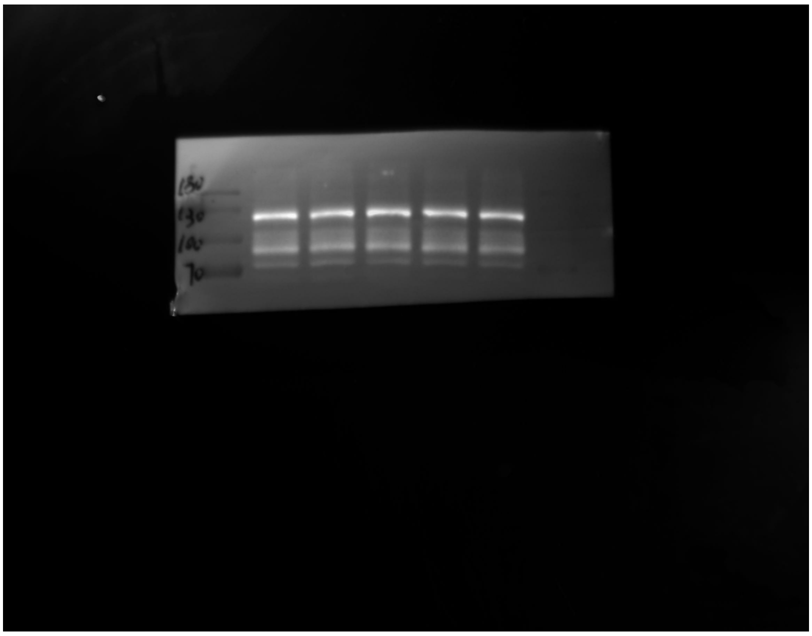

Figure 4 p-ERK

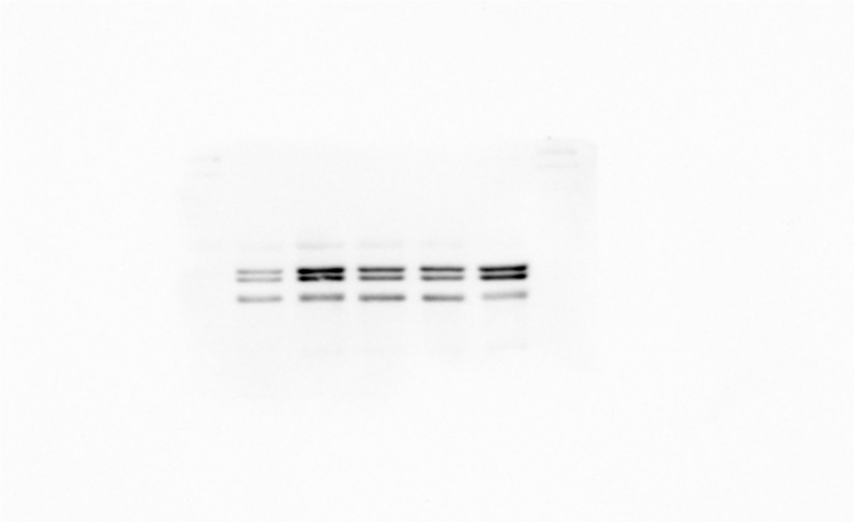

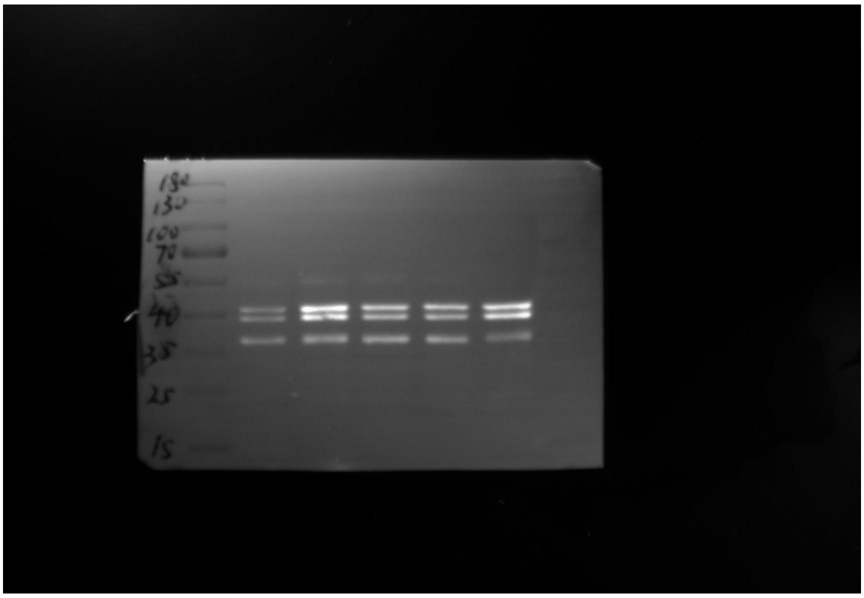

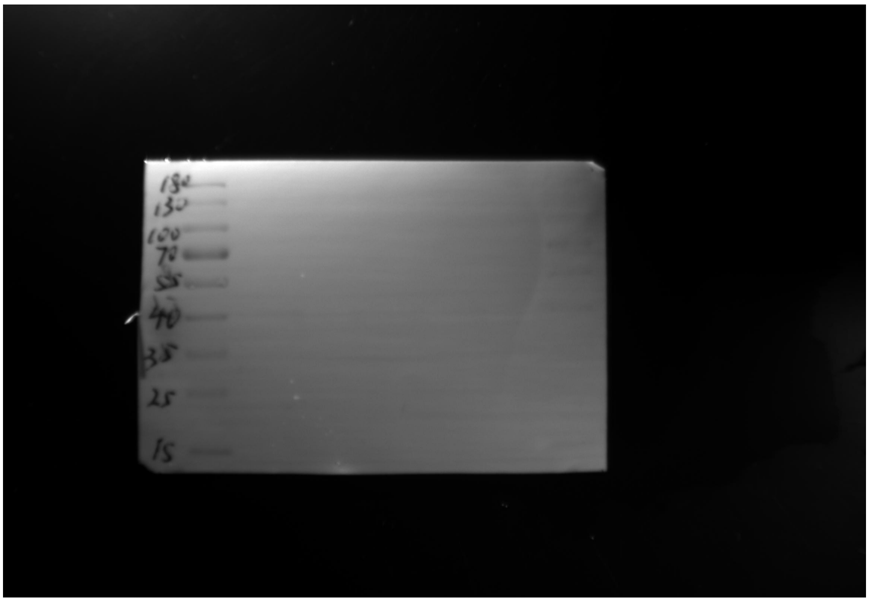

Figure 4 ERK

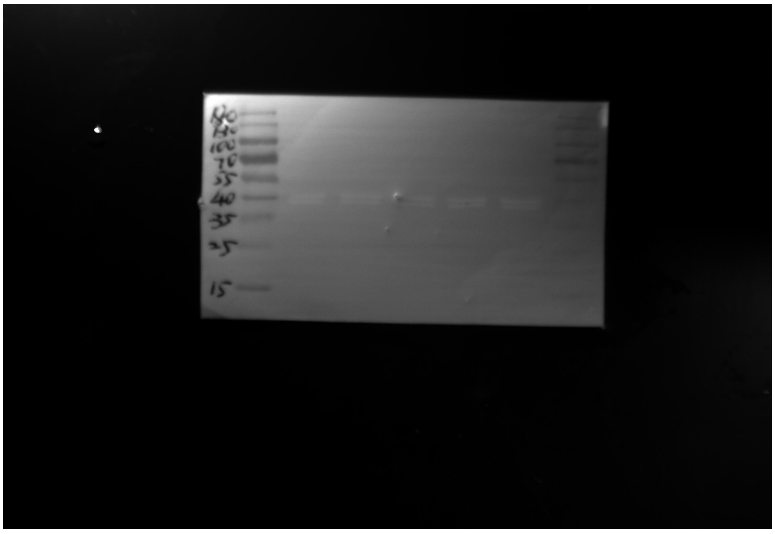

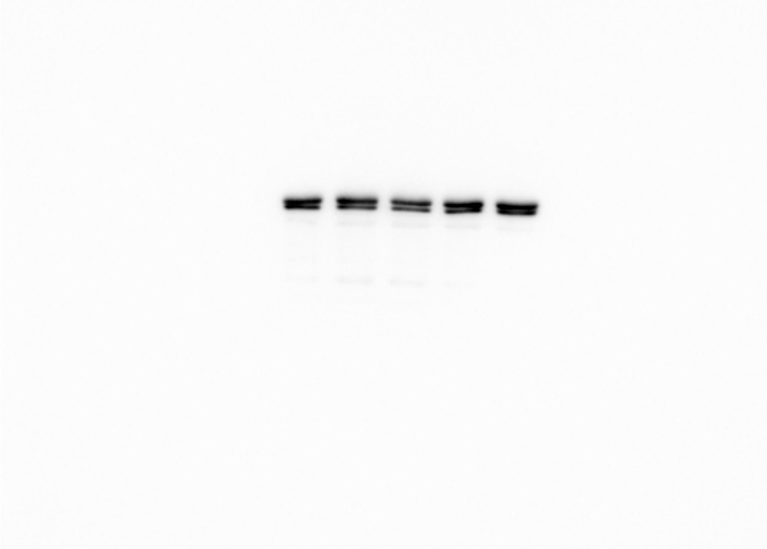

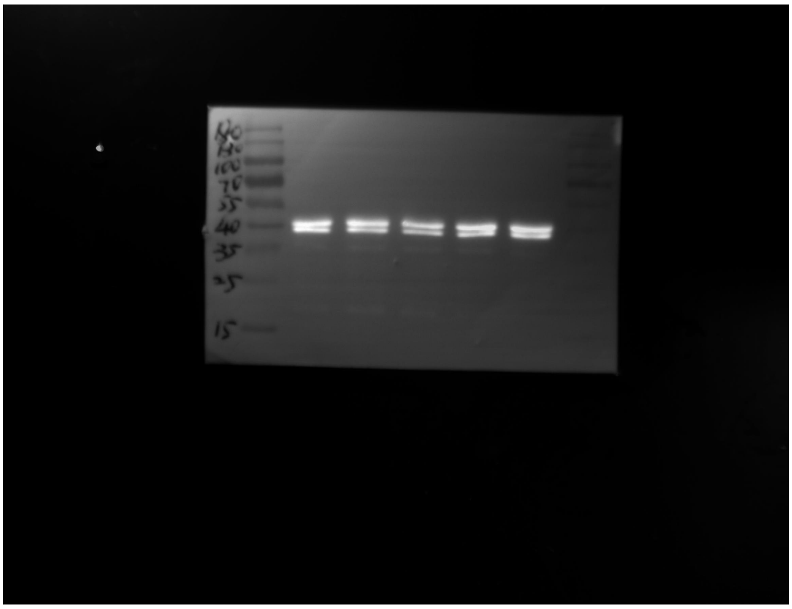

Figure 4 S100A4

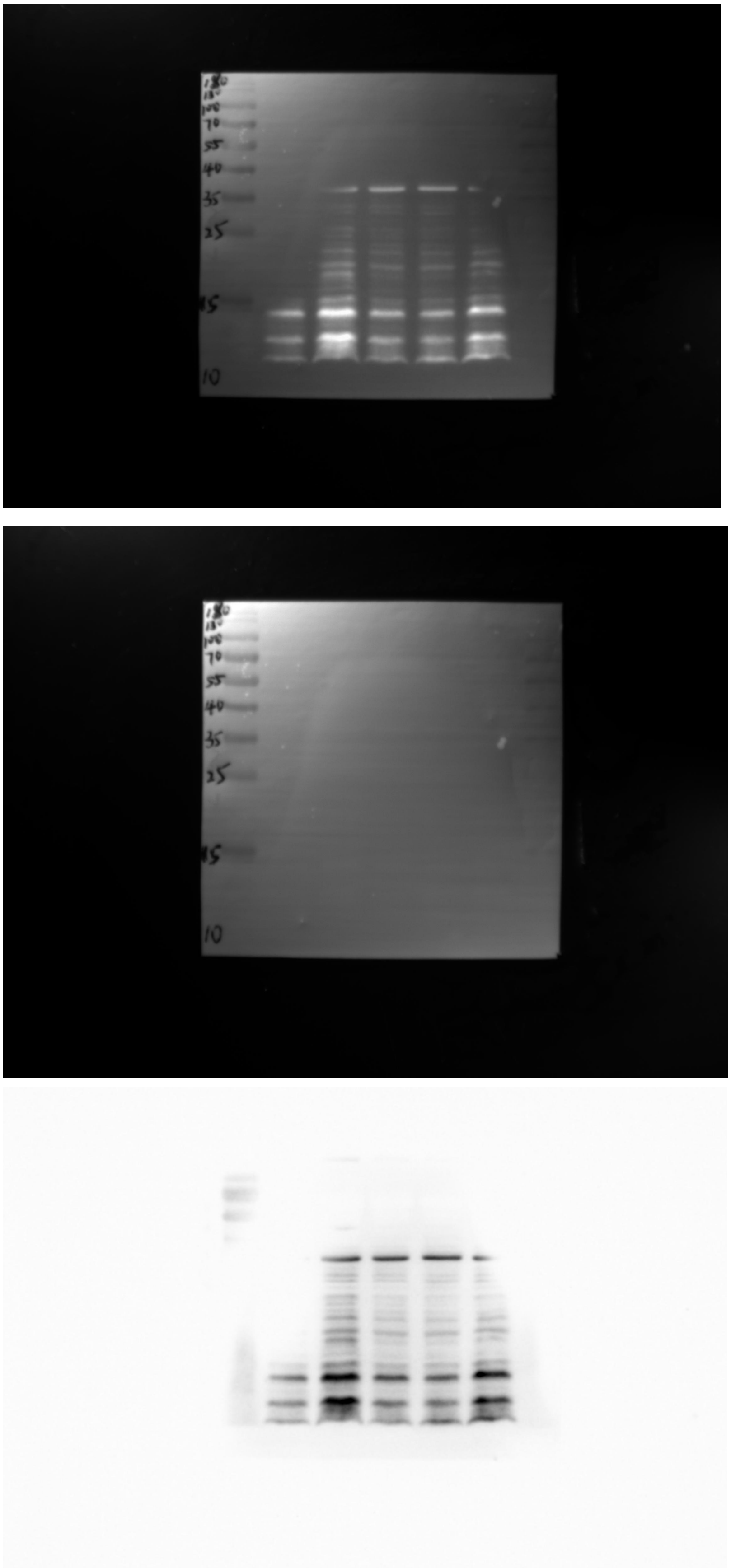

Figure 4 GAPDH

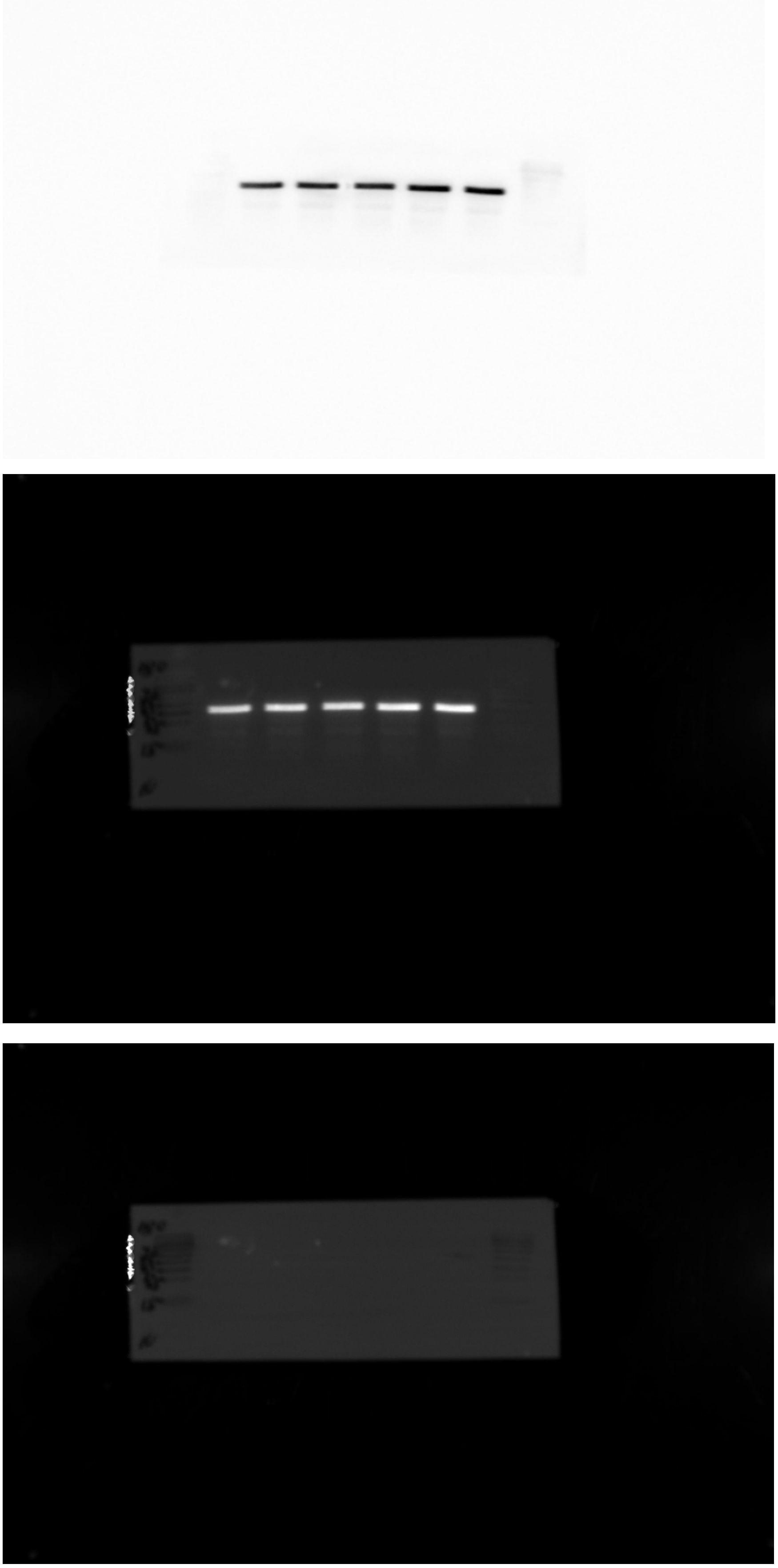

Marker Thermo Fisher

PageRuler Prestained Protein Ladder

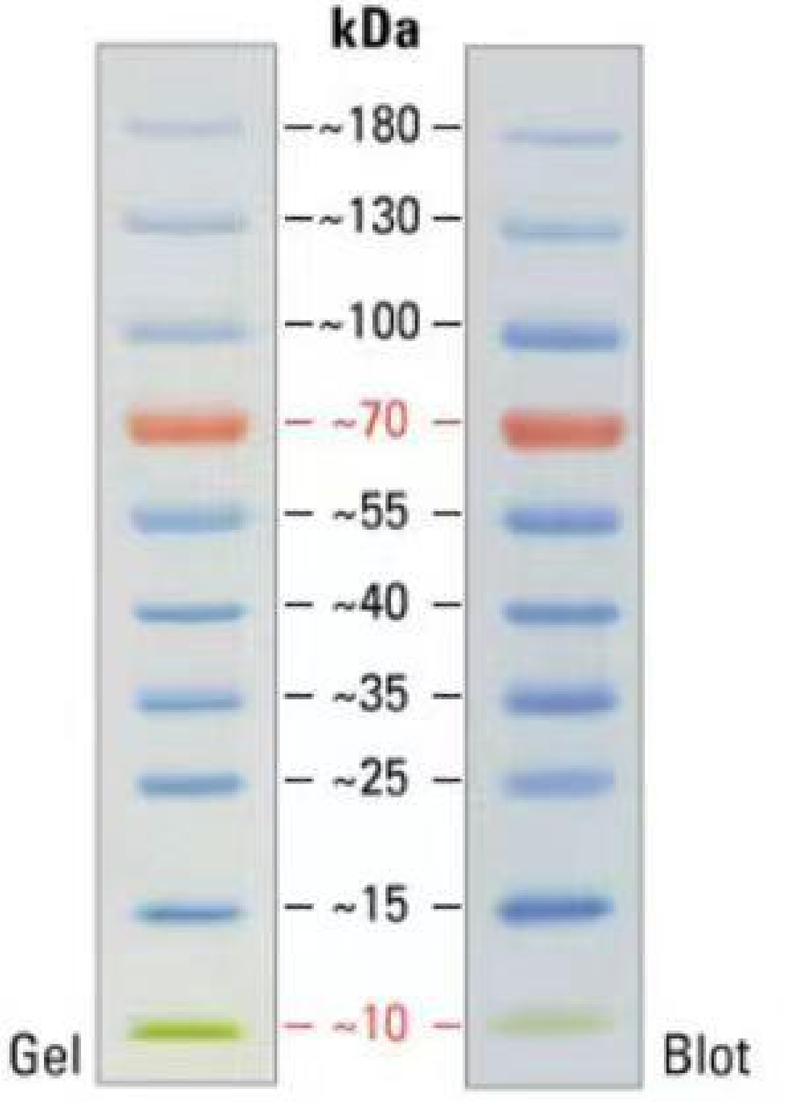

4-20% Tris-glycine SDS-PAGE
